# Transient pulmonary and gastric bleeding after iopamidol administration in a patient with marginal zone lymphoma: a case report

**DOI:** 10.1186/s12890-024-02993-z

**Published:** 2024-04-22

**Authors:** Weixian Xu, Miaozhen Chen, Songtao Liu, Yi Su, Yunhai Zhang

**Affiliations:** 1 The Eighth Clinical Medical College of Guangzhou University of Chinese Medicine, 6 Qinren Road, Chancheng District, 528000 Foshan City, Guangdong Province China; 2 Foshan Clinical Medical School of Guangzhou University of Chinese Medicine, 3 Sanyou South Road, Chancheng District, 528000 Foshan City, Guangdong Province China; 3 Department of Critical Care Medicine, Foshan Hospital of Traditional Chinese Medicine, 6 Qinren Road, Chancheng District, 528000 Foshan City, Guangdong Province China

**Keywords:** Transient hemorrhage, Iopamidol, Marginal zone lymphoma, Case report, Pulmonary hemorrhage, Gastric hemorrhage

## Abstract

**Background:**

Iopamidol is a non-ionic, water-soluble iodine contrast agent that is considered safe for intravenous or intra-arterial administration and is widely used both in the general population and in patients undergoing oncological treatment. While adverse reactions to iopamidol have been documented, to date, no pulmonary and gastric hemorrhages induced by iopamidol have been reported in oncology patients. We report the first case of this complication.

**Case presentation:**

We report the case of a 60-year-old woman with marginal zone lymphoma who was receiving antineoplastic therapy. As part of the investigation for the condition, she underwent chest enhancement CT with iopamidol. Shortly thereafter(within five minutes), she experienced hemoptysis and hematemesis. She was intubated and admitted to the intensive care unit. Pre- and post-contrast images demonstrated the course of the hemorrhage. Flexible bronchoscopy and gastroscopy on the following day showed no active bleeding, and the patient recovered completely after antiallergy treatment. We speculate that contrast-induced hypersensitivity was the most likely cause of the transient pulmonary and gastric bleeding.

**Conclusion:**

Although rare, the complications of iopamidol, which may cause allergic reactions in the lungs and stomach, should be considered.

## Background

Iopamidol, a contrast agent widely used internationally, has been considered safe [1], and hypersensitivity reactions following iopamidol administration occur in approximately 1–3% of patients [2, 3]. The most commonly reported adverse reactions include erythema, urticaria, vomiting, and generalized rashes. The most commonly affected organs and systems are the skin, respiratory system, and gastrointestinal tract. Hypersensitivity reactions to iopamidol are acute in 71.1% of cases and are most commonly Class I [4]. Reports of severe and fatal reactions resulting in death are less common [5]. Transient pulmonary and gastric hemorrhage due to iopamidol allergy has never been reported. Here, we report a case of transient pulmonary and gastric hemorrhage following chest and total abdominal enhancement CT with iopamidol in a woman with marginal zone lymphoma who was receiving antineoplastic therapy.

## Case presentation

**Fig. 1:**
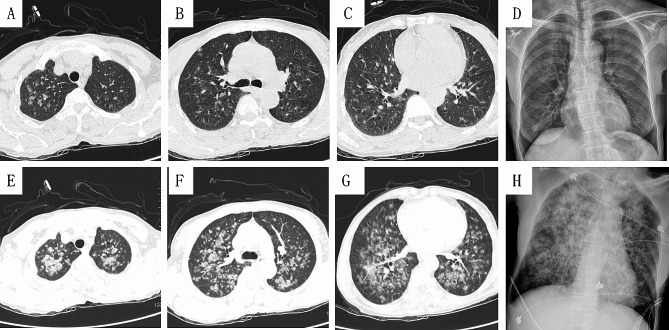
(A-D)10:45:45am, before the injection of iopamidol; (E-G)10:50:05am, after the injection of iopamidol; (H)11:30:00am, After endotracheal intubation

A 60-year-old woman was admitted to our oncology department on 2021-06-21 with recurrent palpitations and findings of anemia for more than 2 months, diagnosed as marginal zone lymphoma and treated with antitumor therapy. Laboratory examinations revealed anemia, renal function damage, hyperuricemia, and chronic viral hepatitis B, and bone marrow puncture results suggested a lymphoma. However, the patient’s platelet count and coagulation, liver, and heart functions were normal, and there was no bleeding. Antinuclear (ANAs), anti-neutrophil cytoplasmic (ANCAs) and anti-glomerular basement membrane (GBM) antibodies were all negative. Complement levels were normal. As part of the examination of her condition, she was required to undergo an enhanced CT examination of the chest and whole abdomen. On the day of the examination, the patient entered the CT room at 10:43 a.m. and an iopamidol syringe was attached without incident. A chest CT was performed at 10:45 a.m. Scattered exudates could be seen (see Fig. 1-ABCD). After a static push of contrast agent, at 10:50 it was shown that the pulmonary exudate was rapidly increasing (see Fig. 1-EFGH). At 10:54, she felt very bloated and had to sit up. At 10:56, She was unconscious after spitting blood. The adjoining room’s emergency physician arrived in the CT room and assisted with resuscitation. Mechanical ventilation was given after endotracheal intubation, and hemorrhagic sputum could be drawn into the airway. An indwelling gastric tube connected to a negative pressure box can induce bloody fluid. At 12:10, the patient became conscious and could move his limbs autonomously. At 14:35, Fibrobronchoscopic alveolar lavage was performed. We saw bloody sputum and several reddish neoplasms in the segmental bronchus. Bronchoscopes at that time used eyepieces, so no photos could be taken. We did not test sputum for the haemosiderin of alveolar macrophages, so Golde score could not be calculated. Despite of the absence of true BAL, we consider an alveolar haemorrhage as probable. She was sent to the ICU for further treatment.

The patient denies a history of food and drug allergies. On physical examination, she had an anemic appearance, and small, 0.5–1 cm, hard, non-tender, and well-defined lymph nodes were palpable in the left axilla and bilateral groins. The remaining superficial lymph nodeswere not palpable. The respiratory sounds in both lungs were slightly coarse, and crackles were heard bilaterally.

The patient was administered levofloxacin for infection, norepinephrine for gastric lavage, esomeprazole for acid inhibition and gastric protection, methylprednisolone sodium succinate for allergy treatment, and.

blood transfusions for improving anemia.(Table 1).

**Table 1:** The results of the patient’s blood routine examination

	Before CT	After CT	reference range	measuring unit
white blood cell, WBC	7.79	14.53$\uparrow$	3.5–9.5	10^9/L
red blood cell, RBC	2.07$\downarrow$	1.79$\downarrow$	3.8–5.1	10^12/L
Hemoglobin, HGB	60$\downarrow$	52$\downarrow$	115–150	g/L
hematocrit, HCT	18.7$\downarrow$	16.8$\downarrow$	35–45	%
blood platelet	157	177	125–350	10^9/L
percentage of neutrophils	46.5	38.6$\downarrow$	40–75	%
percentage of lymphocyte	37.7	52.3$\uparrow$	20–50	%
percentage of monocyte	11.5$\uparrow$	6.2	3–10	%
percentage of eosinophils	3.7	2.2	4–8	%
percentage of basophils	0.6	0.7	0–1	%
neutrophils	3.62	5.61	1.8–6.3	10^9/L
lymphocyte	2.94	7.6$\uparrow$	1.1–3.2	10^9/L
monocyte	0.9$\uparrow$	0.9$\uparrow$	0.1–0.6	10^9/L
eosinophils	0.29	0.32	0.02-0.52	10^9/L
basophils	0.05	0.1$\uparrow$	0-0.06	10^9/L

“$\uparrow$” means higher than the reference range.“$\downarrow$” means lower than the reference range

**Fig. 2:**
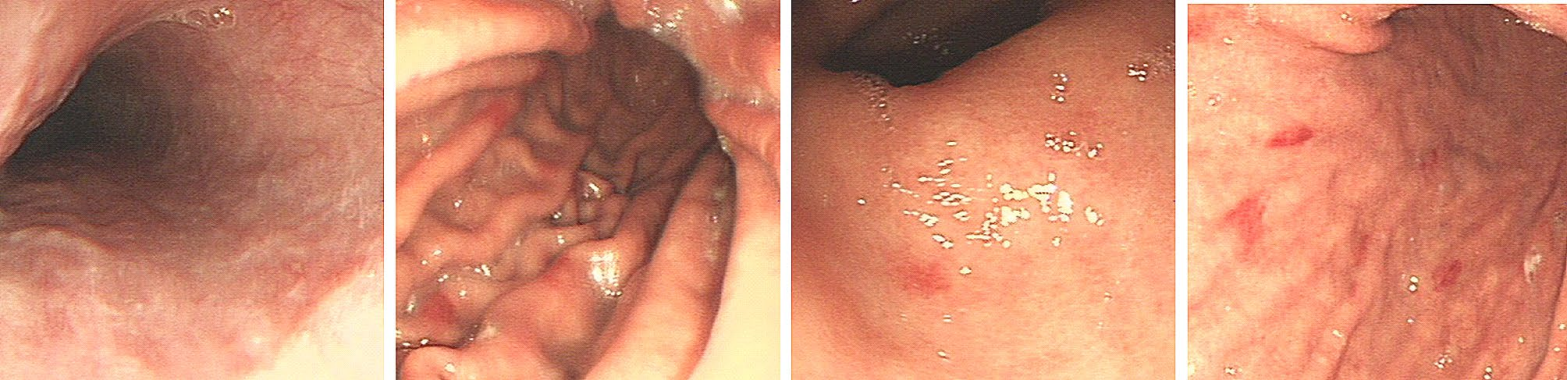
Gastroscopy: mucosal congestion in the body of the stomach, several mucosal haemorrhagic spots in the greater curvature and posterior wall, no active bleeding or ulceration, normal pattern of mucosal folds

On the second day after admission to ICU, gastroscopy showed congestion of the gastric body mucosa, and several mucosal bleeding spots were seen in the greater curvature and posterior wall, without active bleeding or ulceration, and the morphology of mucosal folds was normal, as shown in Fig. 2. Fiberoptic bronchoscopy showed no bleeding, but the patient had a secondary infection after pulmonary hemorrhage. After one week of treatment, the patient was weaned and extubated, and transferred to the general ward.

## Discussion

Pulmonary hemorrhage may be caused by a variety of disorders, and some drugs have also been implicated in the a etiology of diffuse alveolar hemorrhage.On Pneumotox.com site, many publications (see Table 2)are available concerning lung hemorrhage or pulmonary oedema after contrast agents, such as cannabis, abciximab, aspiration, amiodarone, propylthiouracil.

**Table 2:** Cases of pulmonary hemorrhage from Pneumotox.com (By 2024-3-15)

Article	Drug	background disease	Other drugs/ treatment	Possible reasons of lung haemorrhage
Uyar 2009 [19]	5-nitroimidazole	amebiasis	none	PT
Vizzardi 2008 [20]	abciximab	HP, MI, HF	aspirin, heparin.	CD, HF
Turan 2020 [21]	abciximab	HP, MI	clopidogrel, aspirin, heparin.	CD
Seto 2011 [22]	bevacizumab	branch retinal vein occlusion, gastric cancer	none	diminish the regenerative capacity of endothelial cells
Tahir 2015 [23]	alemtuzumab	RF, Alport syndrome,	renal transplantation, methy prednisolone, chlorpheniramine, paracetamol	Sepsis
Ohtsuka 1997 [24]	Propylthiouracil	GD	none	AAV
Yamauchi 2003 [25]	Propylthiouracil	GD, HP, hyperlipidaemia,	Simvastatin, ethy icosapentate, amlodipine besilate	AAV
El-Fakih 2008 [26]	Propylthiouracil	GD	none	AAV
Arai 2018 [27]	methimazole	GD	none	AAV
Perri 2007 [28]	azathioprine	IPF, HP, CKD, bladder cancer	Prednisone, beclomethasone inhalations, nadolol and hydrochlorothiazide	Myelosuppression, thrombocytopenia, pneumonia
Tanawuttiwat 2010 [29]	Amiodarone	AF, HP, AKI, rhabdomyolysis,	intravenous hydration, metoprolol and warfarin	PT, AHF, MI, CD
Saeed 2019 [30]	Amiodarone	MI, HF, COPD, AF, T2DM, HP	none	PT
Mengar 2020 [31]	Cocaine	None(smoking and drinking),	mechanical ventilation, anti-infection	HF
Villeneuve 2020 [32]	“vaped” electronic Cigarettes(nicotine salt)	none	comprehensive treatment	PT
Kalinczuk 2011 [33]	Aspirin, clopidogrel	PFO	none	CD
Kelchen 2013 [34]	aerosol propellant	none	marijuana	PT
Grassin 2011 [35]	cannabis	none	none	PT
Moatemri 2016	cannabis	none	none	PT
Nakamura 2018 [36]	topikku GX(include acetaminophen and chlorpheniramine)	None	None	CD, PT
Ugajin 2020	Iodinated Contrast Medium	CKD, HP, MI, HF	comprehensive treatment	PT, HF, CD*

HP, hypertension; MI, myocardial infarction;AF, atrial fibrillation;HF, heart failure;RF, renal failure;AKI, Acute kidney injury;CKD, chronic kidney disease;GD, Graves’ disease with hyperthyroidism;IPF, idiopathic pulmonary fibrosis;COPD, chronic obstructive pulmonary disease;T2DM, diabetes mellitus type 2;

PFO, patent foramen ovale; AAV, antineutrophi cytoplasmic antibody (ANCA)-associated vasculitis;PT, Pulmonary toxicities;

CD, coagulation disorder; CD*, coagulation disorder cause by antiplatelet or anticoagulant therapy (Necessary for myocardial infarction but not mentioned)

The differential diagnosis of pulmonary hemorrhage is broad. Congestive heart failure, infectious causes, vasculitis, thrombocytopenia, coagulation disorders(primary or secondary) should be considered.To our knowledge, this is the first reported case of transient pulmonary and gastric hemorrhage caused by an iopamidol-induced hypersensitivity reaction. Enhanced CT documented the complete pulmonary hemorrhage. The patient furthermore developed hematemesis at the end of the CT examination on sitting up, and lost consciousness. No further bleeding was observed after antiallergic treatment. Based on the patient’s clinical presentation (bleeding developed, resolved quickly, and basophilia) and treatment outcome (the patient recovered after antiallergic treatment), we determined that this was a type I hypersensitivity reaction.

Learning about the physico-chemical and pharmacological properties of drug is the basis of our medicine [6]. Despite the widespread use of iopamidol, the mechanisms underlying its rapid hypersensitivity reactions remain unclear [7]. However, the most commonly accepted view is that non-ionic contrast agents trigger immunoglobulin E (IgE)-mediated allergic reactions [8–11]. A Type I hypersensitivity reaction is a local or systemic reaction caused by the IgE-mediated release of biologically active mediators from mast cells and basophils, which develops and subsides rapidly. It often causes physiological disturbances with little or no serious tissue or cell damage and has a marked individual and genetic predisposition [12]. We hypothesize that iopamidol acts as a semiantigen that enters the body and acquires immunogenicity by binding to proteins, thereby becoming an allergen. IgE binds to the IgE Fc receptor on the surface of mast cells or basophils via its Fc segment, placing the body in a state of sensitization to iopamidol. This causes capillary dilation and increases vascular permeability, resulting in hemorrhage. Due to limitations of resource constraints or lack of laboratory assays, we couldn’t get more immunological and hypersensitivity tests.

Although studies have shown that skin testing for non-ionic contrast media can help manage immediate hypersensitivity reactions to iodinated contrast media, the specific IgE-mediated mechanisms that may be involved are unclear and are subject to wide variations in positivity rates across national and central study populations, ranging from 4.2–73% [8, 11–17]. Further, in most cases, the skin test results are negative [18]. The instructions also mention that the iodine allergy test does not predict whether a serious or fatal reaction to the contrast agent will occur; therefore, the iodine allergy test is not recommended. However, taking a detailed history prior to contrast administration and focusing on the patient’s history of allergic reactions may be more accurate than allergy testing in predicting potential adverse reactions.

## Conclusion

We believe that clinicians should ask patients about their allergy history before administering contrast media. Patients with a history of contrast reactions, allergy to iodine, or metabolic or hypersensitivity states have a high incidence of adverse reactions and are advised not to undergo iodine contrast testing unless specifically needed. Detailed history-taking and focusing on a patient’s history of allergic reactions and sensitization may be more accurate than allergy testing in predicting potential adverse reactions. The administration of antihistamines or glucocorticoids as prophylactic medications prior to contrast examination may prevent or reduce the incidence of allergic reactions. Allergic reactions to contrast media can be classified as mild, moderate, or severe. Mild reactions are treated symptomatically, whereas moderate and severe reactions should stop the drug immediately and treated by immediate vascular administration.

Finally, the possibility of adverse reactions needs to be considered, not only among radiologists, but also among other healthcare professionals, such as oncologists, ICU doctors, prescribers, and medical staff, who should be pre-trained in first aid measures and prepared with the necessary resuscitation drugs and equipment. Awareness of the safety of contrast agents must be raised, and it is important to treat hypersensitive individuals with a better understanding of susceptibility risk factors, potential interactions, and other relevant aspects.

## Data Availability

Data sharing is not applicable to this report as no datasets were generated or. analyzed during the current study.

## Abbreviations

IgE: immunoglobulin E
